# Interpretability and clinical utility of the strength and stressors in parenting questionnaire

**DOI:** 10.1111/sjop.13073

**Published:** 2024-09-16

**Authors:** Sara Burge, Anna Eva Hallin, Carmela Miniscalco, Anders Sand, Sofia Strömbergsson

**Affiliations:** ^1^ Division of Speech and Language Pathology, Department of Clinical Science Intervention and Technology, Karolinska Institutet Stockholm Sweden; ^2^ Department of Neurology Danderyd Hospital Stockholm Sweden; ^3^ Gillberg Neuropsychiatry Centre Institute of Neuroscience and Physiology, Sahlgrenska Academy, University of Gothenburg Sweden; ^4^ Child and Adolescent Neuropsychiatry Unit Queen Silvia Children's Hospital, Sahlgrenska University Hospital Gothenburg Sweden

**Keywords:** Developmental disabilities, parental stress, parental self‐efficacy, parental engagement, family, family‐centered care, interpretability

## Abstract

This study aimed to enhance the interpretability and clinical utility of the strength and stressors in parenting (SSF) questionnaire, a parent‐reported questionnaire designed to assess strength, stress and associated risks of mental ill‐health in parents of children with developmental disabilities. Responses to the SSF and a demographic questionnaire were collected from 576 parents of children with (*n* = 203) and without (*n* = 373) developmental disabilities. To enhance the interpretability of the SSF, a subset of 129 parents were invited to complete an additional questionnaire consisting of three free‐text questions regarding recent help‐seeking behavior, experiences of mental ill‐health and experiences of parenthood. Parents' responses to the free‐text questions were then categorized as indicative of higher or lower degrees of stress and compared to their SSF score distribution to derive empirical cut‐offs for strength, stress and risk of mental ill‐health as measured by the SSF. The credibility of these cut‐offs was evaluated by comparing the cut‐offs with SSF scores collected from the other 447 parents. Finally, SSF scores from parents of children without developmental disabilities (*n* = 373) were used to generate percentile values for the SSF to enable a standardized interpretation of SSF scores. To increase the utility of the SSF, we examined a recurring pattern of missing answers to items 23 and 33–38, noted in previous studies of the SSF and repeated in the present study. These items were excluded from further analysis since our examination revealed that they were not missing at random but rather constituted real differences in parental experiences, such as receiving a healthcare allowance, or caring for more than one child. The proposed empirical cut‐offs performed well in discriminating between the two groups and yielded a specificity of 77–89% and a sensitivity of 68–76% for the strength, stress and risk of mental ill‐health subscales of the SSF. This study also presents a conversion chart associating each SSF score with a corresponding percentile value. We propose modifications to the SSF, whereby items 23 and 33–38 are excluded, which will enable a more reliable assessment of parental experiences. This will, together with the empirical cut‐offs and percentile values, enhance the interpretability and clinical utility of the SSF.

## INTRODUCTION

Parental stress is distress caused by a perceived imbalance between the demands of parenting and the available resources of the parent (Deater‐Deckard, 2004). While common in parenthood, parental stress nevertheless constitutes a risk of mental ill‐health for the parent, and may have negative effects on parenting behaviors, child development, and the family system (Fang, Luo, Boele, Windhorst, van Grieken & Raat, 2022). Parents of children with developmental disabilities face a heightened risk of experiencing prolonged and elevated parental stress compared to parents of typically developing children (Hastings, 2016; Scherer, Verhey & Kuper, 2019). These parents also face stressors that are unique to the task of parenting children with disabilities (Beckers, Smeets & van der Burg, 2021; Smith & Samuels, 2021). Consequently, healthcare professionals working with children with developmental disabilities and their families need to be well‐informed about parental stress and regularly assess to what extent parental stress is currently impacting parental well‐being (Barroso, Mendez, Graziano & Bagner, 2018), as it may affect not only whether parents themselves need support from healthcare services (Hoyle, Laditka & Laditka, 2021), but also the extent to which parents may be able to engage in interventions aimed at their child (Smith & Samuels, 2021).

Parental engagement refers to the variety of ways in which parents are involved and invested in interventions aimed at their child (Melvin, Meyer & Scarinci, 2020). Parents of children with developmental disabilities may exhibit different levels of engagement in interventions, from initiating contact, to attending sessions, to participating more actively in, and outside of, sessions (Haine‐Schlagel & Walsh, 2015). Parents may also assume different roles throughout an intervention, ranging from more passive to more active, collaborative and independent (Smith & Samuels, 2021). There are many benefits to parental engagement. For one, parental engagement may increase parents' self‐efficacy and improve their understanding of their child's needs (Bode, George, Weist, Stephan, Lever & Youngstrom, 2016). In addition, it may lessen experiences of parental stress (Willemen, Kuzminskaite, Maurice‐Stam, *et al*., 2022). Further, parental engagement is associated with better intervention outcomes for the child (Kuhlthau, Bloom, Van Cleave, *et al*., 2011).

However, there are also risks. High intervention doses and expectations to adhere to strict intervention protocols at home may be time consuming and challenging, causing parents to sacrifice necessary recuperation (Beckers *et al*., 2021). Lack of clarity regarding which role parents are expected to assume in intervention may exacerbate experiences of stress (Smith & Samuels, 2021). If parents perceive an imbalance between healthcare professionals' expectations of parental engagement and their own perception of available resources, the intervention in itself may constitute a stressor, resulting in therapy‐related parental stress (Beckers *et al*., 2021).

Historically interventions aimed at children with developmental disabilities have been primarily child‐centered (Calder, Ward, Jones, Johnston & Claessen, 2018). However, recent decades have seen a shift towards family‐centered interventions, driven in part by research on the importance of family‐centered care to promote both parental well‐being, and satisfaction with care as well as better intervention outcomes for children with developmental disabilities (Klatte, Ketelaar, de Groot, Bloemen & Gerrits, 2024; Kuhlthau *et al*., 2011). Nevertheless, this shift in focus is not always reflected in the choice of outcome measures (Calder *et al*., 2018). Since family‐centered interventions may entail higher expectations of parental engagement (Kokorelias, Gignac, Naglie & Cameron, 2019; Smith & Samuels, 2021) potentially elevating the risk of therapy‐related parental stress (Beckers *et al*., 2021) this raises the need for a wider set of outcome measures that enable healthcare professionals to better evaluate the effect of interventions not only on the child, but also on the parents, and the family system (Calder *et al*., 2018; Popov, Phoenix & King, 2021). While there are many instruments designed to measure aspects of parental functioning, fewer are designed to capture the experiences of parents of children with disabilities. Among the most widely used instruments in research on parents are the parental stress index (PSI) (Abidin, 1983), and the parenting stress scale (PSS) (Berry & Jones, 1995). Both, however, are designed to measure general parental stress rather than parental stress in parents of children with developmental disabilities.

The primary objective of this study is to enhance the interpretability (Mokkink, Terwee, Patrick *et al*., 2010) and clinical utility (Badrick & Bowling, 2023) of one instrument commonly used in Sweden for measuring parental strength, stress, and risks associated with mental ill‐health in parents of children with developmental disabilities; the strengths and stressors in parenting (SSF) questionnaire. Originating from the American family impact questionnaire (FIQ) (Donenberg & Baker, 1993), the SSF has undergone translation, cultural adaptation and further development and has previously been published on in two different master theses (Cederblad, 2013; Falck & Ternert, 2016), and in an evaluation of its structural validity (Ivarsson, Danielsson, Andersson, Gothilander & Granlund, 2023). The SSF has been used as an outcome measure in the national quality registry for habilitation services (HabQ), in research studies, such as the child participation and mental health (CHILD‐PMH) study and clinically in the Swedish habilitation services (Ivarsson *et al*., 2023) that cater for children with formal diagnoses of autism, intellectual disability (ID), or physical disability.

However, the potential for clinical use of the SSF may be greater. A large group of children with other developmental disabilities, such as developmental language disorder (DLD), attentional deficit hyperactivity disorder (ADHD), or children with yet undiagnosed developmental disabilities, are seen in primary care by a range of healthcare professionals, such as speech language pathologists, psychologists, occupational therapists, physiotherapists, and dieticians. For this group of children, and their families, there is currently no widely used and accepted instrument for assessing parental strength, stress, and risk associated with mental ill‐health.

Compared to similar instruments, the SSF has several advantages, such as its wide use in both clinical practice and research, its coverage of both positive and negative impact on the parent across multiple domains of parenting, and not least the fact that it contains items that specifically apply to the experiences of parents of children with disabilities, such as the degree of help offered from healthcare services. Nevertheless, the SSF has two prominent weaknesses that impact its interpretability, and hence its potential for more widespread utilization in healthcare services aimed at children with developmental disabilities, and in research. First, in its current form, the SSF contains questions that are only relevant to certain subgroups of respondents. To enhance the overall clinical utility of the SSF, it would be advisable to either modify or eliminate those questions. Second, the interpretation of SSF scores remains undefined. While higher scores on individual subscales likely correspond to more strength, higher stress levels, or an elevated risk factor, it remains to be determined what constitutes “typical values” or values indicative of being at risk. Our objective is to address and improve these aspects of the SSF. In doing so, the clinical utility of the SSF will be enhanced, both in initial evaluations and as an instrument to measure progress.

### Aims

The overarching aim of this study is to increase the interpretability and clinical utility of the SSF through four specific aims:

1. Identify and suggest a strategy for handling items that are only relevant for a subset of parents. This will result in a more widely usable and precise survey tool.
2. Provide an interpretation for SSF scores by deriving empirical cut‐offs for being “at risk of mental ill‐health.” This will imbue SSF scores with practical and clinical significance.
3. Test how such empirical cut‐offs align with SSF scores from parents of children with and without developmental disabilities. This will determine the credibility of our cut‐offs.
4. Provide percentile values for SSF scores from parents of children without developmental disabilities. This will contribute to a more comprehensive understanding of parental experiences of well‐being.

## METHODS

### Participants

**Fig. 1:**
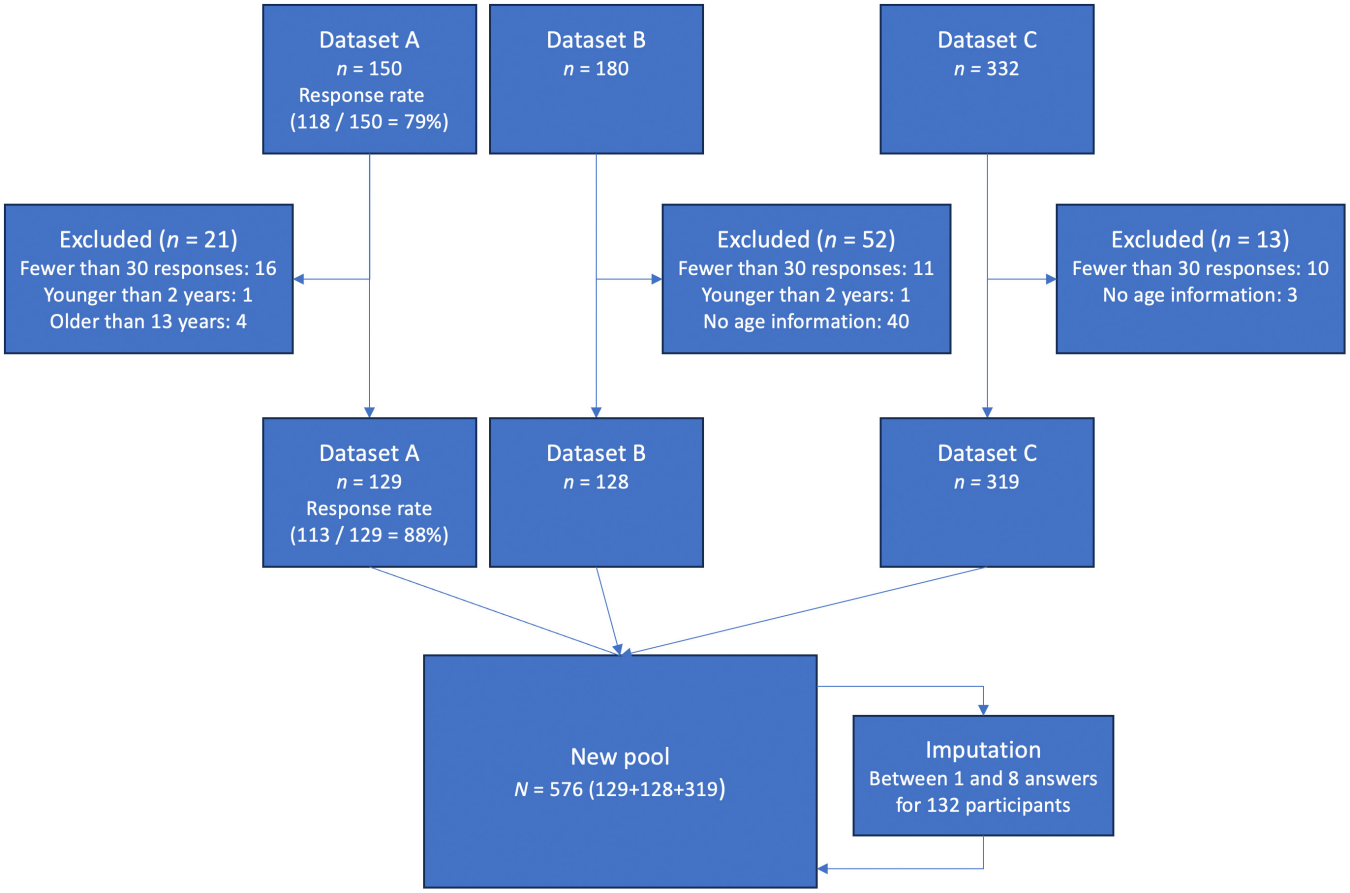
Flowchart of the assembly of the combined dataset.

In this study, we collected two datasets (dataset A, *n* = 150, and dataset B, *n* = 180) in the fourth quarter of 2022 and the first quarter of 2023, and combined these with a third preexisting dataset (dataset C, *n* = 332), collected in 2016 (Falck & Ternert, 2016) to increase the sample size and hence the statistical power of our analysis. Combined, the three datasets included responses from 662 respondents, all of whom were parents of children aged 2–13 years, who had initiated the surveys. However, we excluded 86 respondents who either answered fewer than 30 of the SSF questions, did not provide their child's age, or had children who were either too young (under 2 years) or too old (over 13 years) for the scope of the present study. Figure 1 shows a flow‐chart detailing how the pooled dataset was created from the three datasets. In the end, our study included 576 respondents, distributed as follows: *n* = 129 from dataset A, *n* = 128 from dataset B, and *n* = 319 from dataset C. Table 1 offers descriptive statistics for both parents and their children across the various datasets. For a more detailed description of the distribution of age and disabilities across the three datasets we refer to Tables S1, S2, S3 in the supplementary materials.

**Table 1:** Descriptive statistics derived from parent report

	Dataset A *n* = 129	Dataset B *n* = 128	Dataset C *n* = 319
Child disability status *n* (%)			
No disability	89 (69%)	79 (62%)	205 (64%)
Developmental language disorder (DLD)	35 (27%)	45 (35%)	N/A
Autism	9 (7%)	8 (6%)	63 (20%)
Intellectual disability	1 (1%)	1 (2%)	21 (7%)
Other	9 (7%)	8 (6%)	30 (9%)
Child age, median (min‐max)	5 (2–12)	5 (2–13)	10 (6–13)
Parent age, median (min‐max)	38 (27–57)	37 (27–52)	42 (27–68)
Parent educational level *n* (%)			
High school	1 (1%)	0 (0%)	14 (4%)
Post‐secondary education	128 (99%)	128 (100%)	305 (96%)
Child living situation *n* (%)			
Single parent household	8 (6%)	9 (7%)	46 (14%)
Dual parent household (Non‐intact family)	2 (2%)	2 (2%)	27 (8%)
Dual parent household (Intact family)	119 (92%)	116 (91%)	246 (78%)
Siblings in family *n* (%)	102 (79%)	106 (83%)	296 (93%)

*Note*: For child disability status, “Other” includes motor impairments, ADHD, visual and/or hearing impairments.

Among these 576 respondents, 444 (77%) provided complete responses to all the included SSF questions. For the remaining 132 respondents with missing answers on the included SSF questions, 50 respondents had only one missing response, the median number of missing responses was two, and the maximum number of missing responses was eight. To address these missing responses, we employed a stochastic regression imputation based on the answered SSF questions, using the “mice” package in *R* with a single imputation iteration (van Buuren & Groothuis‐Oudshoorn, 2011).

### Dataset A “free text” dataset

Participants in dataset A were recruited via public advertisements on Facebook and targeted advertisements in Facebook groups for parents of children with developmental language disorder (DLD). Parents were then invited to complete an anonymous online survey through the Research Electronic Data Capture (REDCap) data management and survey tool hosted at CLINTEC Karolinska Institutet (Harris *et al*., 2019; Harris, Taylor, Thielke, Payne, Gonzalez & Conde, 2009). The survey consisted of a demographic questionnaire mirroring the demographic data collected in the study by Falck and Ternert (2016) and a digital version of the SSF. Following completion of these surveys, participants could choose to either conclude the survey or continue to a third survey containing subjective and free‐text questions regarding mental health.

### Dataset B

Dataset B was collected using the same recruitment and survey procedure as dataset A with the exception that the survey did not include the optional free‐text questions at the end.

### Dataset C “Falck and Ternert” dataset

To expand the data with parents of older children with and without developmental disabilities we utilized a sample of 332 parents previously reported on in a master thesis work by Falck and Ternert (2016). Parents of children with and without developmental disabilities were contacted by distribution of paper questionnaires to four schools in Västerbotten County in the north of Sweden. Parents of children with developmental disabilities were contacted by distribution of paper questionnaires via the child‐ and youth‐habilitation services in the same county.

### Ethical considerations

Before being presented with any questions, potential respondents in Dataset A and B were presented with written information regarding the nature of the survey and that no responses could be used to identify specific individuals. They then explicitly consented to these conditions to proceed to the survey proper (see Materials, below). Dataset C was collected in a similar manner, as presented in Falck and Ternert (2016), and then graciously shared with the current research team. All data was fully anonymous, as no personally identifiable information was collected or handled in the study. No information could indirectly be traced back to specific individuals, also not via respondents' IP addresses, as these were not registered. As such, the study was not subject for approval by the Swedish Ethical Review Authority. All data was handled in accordance with tenets in the declaration of Helsinki.

### Materials

#### Demographic questionnaire

All parents in datasets A and B were invited to complete a demographic questionnaire via REDCap (Table 1). In addition to demographic information about child and parent age, child living situation and parent education level, parents were also asked to report whether their child had any disability, such as DLD, Autism, intellectual disability, ADHD, or motor, hearing, or visual impairment. Hence, all information on child disability in this study is derived from parent reports.

#### The SSF questionnaire – items, domains, and subscales

The SSF is a self‐rated parent questionnaire consisting of 45 items with a minimum score of 0 and a maximum score of 129. Items 1–43 are graded on a four‐point Likert scale ranging from 0 (“not at all”) to 3 (“very much”). These items aim to assess self‐perceived strength, stress, and risk of mental ill‐health across six different domains, described in Ivarsson *et al*. (2023) as parent's feelings and attitudes, social life, family finances, relationship to the other parent, siblings, and professional support. The remaining two items, items 44–45 consist of global ratings of strength and stress, respectively (Ivarsson *et al*., 2023). There are three subcales to the SSF; Strength (0–67), Stress (0–82) and Risk of mental ill‐health (0–47) and each of the six domains contains items belonging to each of the three subscales (Broberg, 2018). The risk subscale is compounded by items from the stress subscale and item 45 from the strength subscale, constituting the parents' global rating of strength (Broberg, 2018).

#### Modifications to the SSF questionnaire

In our initial examination of the data, we noticed a disproportionate amount of missing answers to items 23 and 33–38 in all of the datasets. In dataset A, only 4% of respondents had answered SSF item 23 and only 67% had answered any of items 33–38. A similar pattern of missing answers was found in dataset B with response rates of 7% and 67% for item 23 and items 33–38 respectively. In Dataset C the response rates were somewhat higher (32% and 86% for item 23 and items 33–38), possibly because this dataset was comprised of parents of slightly older children, some of whom were receiving habilitation services. We then checked reports of missing answers in a previous study of the measurement properties of the SSF by Ivarsson *et al*. (2023) where we again found a similar pattern of missing answers, albeit less pronounced, with an 18.49% mean number of missing answers for items 23 and 33–38, compared to a 2.49% mean number of missing answers to the rest of the SSF items (Ivarsson *et al*., 2023, p. 3) in a dataset consisting entirely of parents of children receiving habilitation services.

This prompted us to conduct a closer review of the items in question which led us to the conclusion that missing answers to these items could only reliably be interpreted as being of the type not missing at random (NMAR) (Bennett, 2001). For example, item 23: “The healthcare allowance constitutes a valuable financial contribution,” within the family finances‐domain can only be answered by parents who receive a healthcare allowance. The other six items (items 33–38), constitute the entire siblings‐domain and address the parent's perception of how parenting the child in question impacts on the child's siblings. These items can only be answered by parents who care for more than one child. Parents who either do not receive a healthcare allowance or who only have one child cannot answer these questions. Hence the large number of missing answers to these items repeated in four distinct data sets is most likely a consequence of actual differences in parental circumstances which generally precludes imputation (Bennett, 2001).

A further argument in favor of excluding these items from the SSF scoring was that items 23 and 36 did not contribute to the factor structure of the SSF in a confirmatory factor analysis conducted in a previous study of the SSF, which led the authors to recommend that these items should be excluded in future calculations of factor scores (Ivarsson *et al*., 2023). Consequently, excluding items 33–38 (constituting a single factor, the *sibling* factor) and item 23 would not hamper use of the factor structure in future studies.

Following these considerations, the choice was made to consistently exclude responses to these seven items when calculating the stress (items 34–35, 38), strength (items 23, 33, 36–37) and risk subscales (items 35, 38). After the exclusion of these items, the potential range for the subscales becomes 0–55 for strength, 0–73 for stress, and 0–41 for risk of mental ill‐health. This modification was done to ensure that the SSF remains an equitable tool for assessing parental experiences across diverse contexts.

#### Free text questions on parental well‐being

Free text questions included in dataset A (“free text”) were presented subsequently to the demographic and SSF questions. The first two questions were yes/no questions, allowing the option of providing free text answers in case of a “yes” response. The questions were (paraphrased into English):

1. Looking back over the past month. During that time, have you sought, or repeatedly contemplated seeking, support from healthcare services because you felt that you lacked the resources to meet the demands of parenting?
  - If yes: can you elaborate on how you felt on these occasions?
2. Over the past month, have you repeatedly experienced signs of mental ill‐health related to parenting?
  - If yes: can you elaborate on how you felt on these occasions?
3. Describe, in your own words, your experiences of parenting during the past month. Consider strengths, challenges, stress, and well‐being.

### Analysis

#### Categorizing parent self‐reports of well‐being

Our operational criteria for identifying parents “at risk of mental ill‐health” was contingent upon parents' self‐reports to the three free‐text questions. Out of the 129 respondents invited to answer these questions, 113 responded to questions 1 and 2 (88%), and 65 responded to question 3 (50%). Seventeen of the responding parents had children with developmental disabilities. Two independent assessors (the author SB and a trained SLP student) read the free‐text responses to question 3 separately, and, without conferring, or looking at any other information (such as the SSF answers, child‐status, or question 1 and 2 above), labeled each respondent as being affected by parental stress “Affected_freetext_” or not. Out of the 65 free‐text responses, the two assessors agreed on their categorization in 52 of the cases (80% exact agreement); the remaining 13 responses were categorized after a consensus discussion between the two assessors. Respondents were labeled as being more, less, or not affected by parental stress based on their replies to questions 1–3. Replying “yes” to any of questions 1 or 2 was used to label the parents as being “more affected,” regardless of their response to question 3. Among those parents who were not “more affected,” parents who were labeled “affected freetext” were then categorized as “less affected.” The remaining respondents who had responded to question 1–3 were labeled as “not affected.” The result of this rather elaborate coding scheme is illustrated in Table S4. In the end 34/65 respondents were categorized as “affected” and 31/65 as “not affected” by parental stress.

### Statistical analyses

To assess the discriminative capacity of the SSF scales in distinguishing between respondents categorized as “not affected” or “more affected” we employed receiver operating characteristic (ROC; Green & Swets, 2000) analysis. Our choice of an empirical cut‐off was informed by a comprehensive evaluation of sensitivity, specificity, and likelihood ratios associated with the ROC curves. Since experiences of parental stress are subjective and internal states (Craig, Operto, De Giacomo *et al*., 2016; Deater‐Deckard, 2004), making the individual parent the most reliable source of information (Guyatt, Osoba, Wu, Wyrwich & Norman, 2002), we prioritized a high degree of specificity over a high degree of sensitivity.

To determine the credibility of the empirical cut‐offs established from dataset A “free text,” we performed a comparative analysis to gauge how the empirical cut‐offs aligned with the SSF scores of parents of children both with and without developmental disabilities obtained from datasets B and C. We conducted a descriptive and graphical examination of SSF scores, and assessed the proportion of respondents scoring above and below these cut‐offs across parent groups and datasets.

To gain a more comprehensive understanding of parental experiences of strength, stress as measured by the SSF, we calculated an empirical cumulative distribution function for the various SSF scales. A priori (before looking at the data) we anticipated that parents of children with disabilities would experience higher stress levels and fewer strengths compared to parents of children without disabilities. Since experiences of stress and strengths may vary depending on the specific disability of the child (e.g., mobility impairment versus autism) we aimed to provide percentile values for parent of children without disabilities. We then used the resulting cumulative distribution function to construct a “conversion chart” converting each specific SSF score to its corresponding percentile score (Table S5). All statistical analyses were conducted using R statistical software (R Core Team, 2021).

## RESULTS

### Deriving empirical cut‐offs for being “at risk of mental ill‐health”

**Fig. 2:**
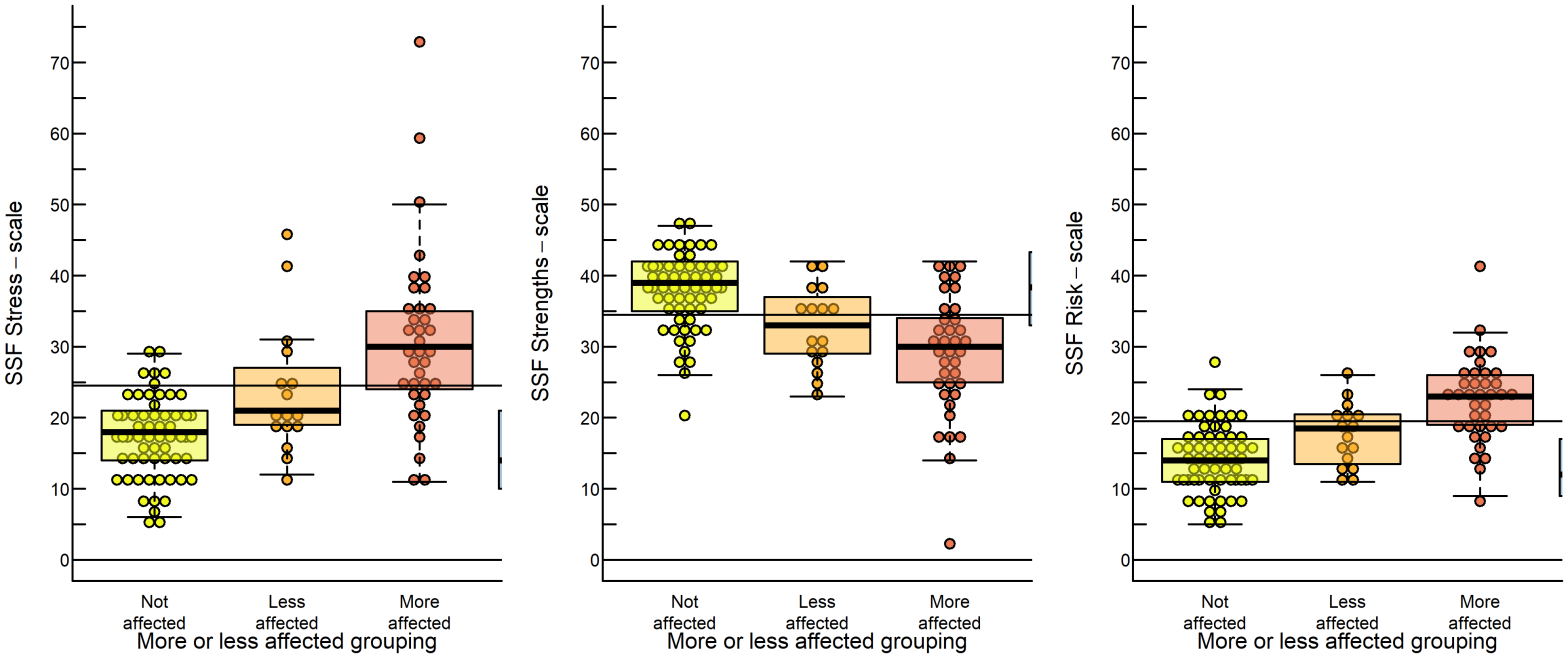
Parents' SSF scores sorted according to being “more or less affected.” Individual parents are shown as individual circles and superimposed are boxplots (illustrating quartiles). The horizontal black line illustrates the suggested cut‐off listed in Table 3. *N* = 129.

In dataset A, parents identified as “more affected” based on their free‐text responses had considerably higher SSF scores for stress, risk, and lower scores for strengths compared to “not affected” parents (Table 2). Figure 2 visually confirms these group differences.

**Table 2:** Descriptive statistics (mean ± SD [lower limit, upper limit of a 95% confidence interval around the mean]) for the different SSF‐scales for the sorting of respondents into being more or less affected by parental stress

SSF‐scale	Not affected (*n* = 61)	Less affected (*n* = 15)	More affected (*n* = 37)
Stress	18 ± 6 [16, 19]	24 ± 9 [18, 29]	31 ± 12 [27, 35]
Strengths	38 ± 5 [37, 39]	33 ± 6 [30, 36]	29 ± 9 [26, 32]
Risk	14 ± 5 [13, 16]	18 ± 5 [15, 20]	23 ± 6 [21, 25]

*Notes*: Only a subset of respondents (*n* = 129) had the possibility of answering Q1–Q3 and thus be sorted as being “more or less affected.” The response rate to Q1–Q3 among those 129 was 88%.

A ROC analysis showed that all three SSF scales performed admirably in discriminating between the two groups, as evidenced by their area under the curve (AUC) values with 95% confidence intervals (Delong) ranging between [0.78, 0.94], [0.73, 0.90] and [0.79, 0.94] for the SSF stress, strengths, and risk scales. After a thorough examination of sensitivity, specificity, and likelihood ratios corresponding to the ROC curves (Figure S1), we opted for a cut‐off point of 25 for stress, 35 for strength, and 20 for risk. The aim was to get as high specificity as possible while retaining an acceptable sensitivity, which meant that cut‐offs yielded a higher specificity (77–89%), and a slightly lower sensitivity (68–76%). Table 3 shows the suggested cut‐off values, along with their associated sensitivity, specificity, and likelihood ratios. The score distribution around the cut‐offs is visually indicated in Fig. 2.

**Table 3:** Suggested cut‐offs on the different SSF scales. These cut‐offs are selected because they are closest to 75% sensitivity in identifying respondents as “more affected,” as compared to “not affected”

SSF‐scale	Cut‐off	Sensitivity	Specificity	LR+	LR−
Stress	≥25	73%	89%	6.36	0.31
Strengths	≤35	76%	77%	3.3	0.32
Risk	≥20	68%	84%	4.12	0.39

*Notes*: Higher values on the stress and risk scale indicate being more affected, whereas the opposite is true for the strengths scale. Thus, a strength score at or below 35 indicates being “more affected.” Sensitivity = percentage of “more affected” respondents being sorted as such. Specificity = percentage of “not affected” respondents being sorted as such. LR+ = positive likelihood ratio; LR− = negative likelihood ratio.

### Testing the empirical cut‐offs against known groups of parents

**Fig. 3:**
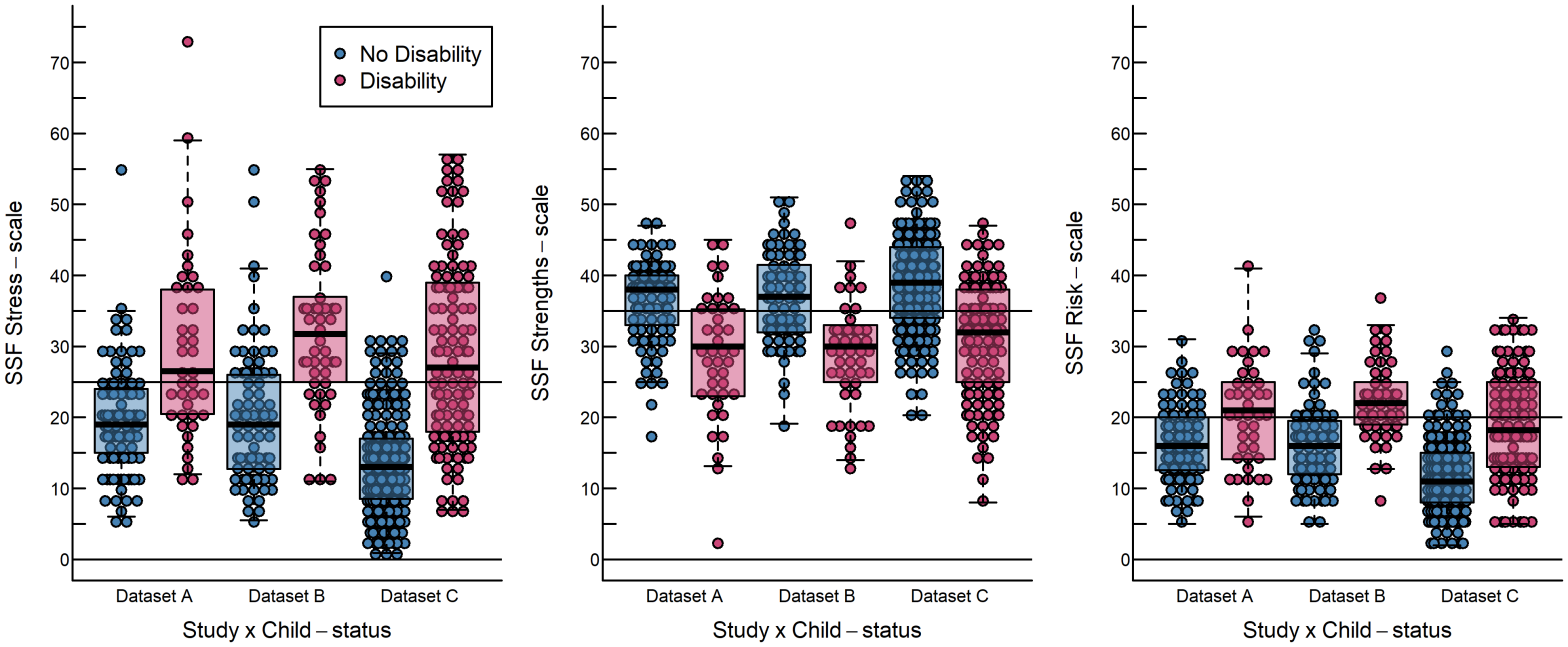
A boxplot diagram of SSF score distribution of known groups in all three datasets. The horizontal black line illustrates the suggested cut‐offs listed in Table 3.

In datasets B and C, we compared pooled data of SSF scores for parents of children with and without developmental disabilities to test the credibility of the empirical cut‐offs. Table 4 presents an overview of SSF score statistics for all participants across the three datasets, along with the proportion of parents scoring above or below our suggested cut‐off values. Figure 3 provides a graphical representation of these comparisons in a boxplot diagram. Across all datasets, parents of children with disabilities experienced higher levels of stress, fewer strengths, and were at greater risk of mental ill‐health compared to parents of children without disabilities. Correspondingly, a larger proportion of parents of children with disabilities exceeded the empirical cut‐offs, represented in Fig. 3 as horizontal lines. In datasets A and B approximately one‐third of parents of children without disabilities exceeded the cut‐offs, while roughly two‐thirds of parents of children with disabilities did so.

**Table 4:** Mean ± SD (lower limit, upper limit of a 95% confidence interval around the mean) for parents of children with developmental disability (DD) or with no disability in the three datasets. Percentage of parents who were sorted above the cut‐off in each dataset

	Stress		Strengths		Risk	
Dataset A: No	20 ± 8 [18, 21]	24%	36 ± 6 [35, 38]	35%	16 ± 5 [15, 17]	27%
Dataset A: DD	30 ± 13 [25, 34]	58%	29 ± 9 [26, 32]	70%	21 ± 7 [18, 23]	58%
Dataset B: No	20 ± 10 [18, 22]	33%	37 ± 6 [35, 38]	43%	16 ± 6 [15, 18]	25%
Dataset B: DD	32 ± 11 [29, 36]	78%	29 ± 7 [27, 31]	84%	22 ± 6 [21, 24]	69%
Dataset C: No	14 ± 7 [13, 15]	9%	39 ± 7 [38, 40]	27%	12 ± 5 [11, 12]	8%
Dataset C: DD	29 ± 13 [26, 31]	57%	31 ± 8 [29, 32]	66%	19 ± 7 [18, 20]	46%

Dataset C exhibited some variations in comparison to the other datasets. Most notably, parents of children without disabilities in this dataset displayed better scores on the respective SSF scales, and a considerably smaller proportion of parents exceeded the empirical cut‐offs (see Fig. 3). Delving deeper into this finding, we considered the demographic differences reported in Table 1, and presented in greater detail in Tables S1, S2, S3, particularly the fact that children in datasets A and B were predominantly younger. As a result, we explored whether parents' SSF scores correlated with child age.

Among parents of children without disabilities, stress scores exhibited a negative correlation with age (*r* = −0.31, *p* < 0.001), Strength scores showed a positive correlation with age (*r* = 0.10, *p* = 0.056), and Risk scores were negatively correlated with age (*r* = −0.33, *p* < 0.0001) across all three datasets. In other words, parents of children without disabilities caring for older children tended to score “better” on the SSF scales. In contrast, among parents of children with disabilities, SSF scores and the proportion of parents exceeding the cut‐offs were relatively consistent across all three datasets and none of the SSF scale scores were strongly correlated with age (correlation coefficients ranging between −0.02 to −0.09, with *p*‐values between 0.18 and 0.80). Thus, among parents of children with disabilities, child age did not significantly influence how they scored on the SSF scales.

In summary, the empirical cut‐offs derived from the “free text” dataset demonstrated reasonably consistent alignment with parents of children both with and without disabilities in two other datasets collected on separate occasions with the exception that parents of older children without disabilities scored lower on the Stress scale.

### Calculating percentile values for SSF scores

Based on the combined datasets A, B, and C, we computed an empirical cumulative distribution function for the various SSF scales among parents of children without disabilities (*n* = 373). Initially, we explored these functions separately for different age groups, given the observed correlation between SSF scores and the child's age. However, as the variations were minimal, and for practical simplicity, we present here the resultant function encompassing all age groups. Upon visual examination of the cumulative distribution function (Figure S2), it closely resembled that of a perfect normal distribution, affirming the suitability of our chosen function. Utilizing this resultant function for parents of children without disabilities, we constructed a “conversion chart,” detailed in Table S5, that associates a specific SSF score with a corresponding percentile score thus enabling users of SSF to determine the percentile corresponding to a given SSF value. Further guidance on how to effectively use this table is provided in the table notes and in the discussion.

## DISCUSSION

The overarching aim of this study was to enhance the interpretability and clinical utility of the SSF to support its future use in clinical practice and research. As for interpretability, we found that scores on the SSF align well with how parents describe their current experiences of stress and well‐being. The reported parental experiences were further used to establish clinical cut‐offs for the subscales of the SSF. In addition, we found that the SSF successfully captured differences between groups of parents that are known to differ in parental well‐being. With this finding as a knowledge base, we calculated percentile values for the SSF based on the SSF scores of parents of children without disabilities, thus providing a common metric for assessing parental well‐being in parents of children with disabilities. Together, these findings can aid the interpretation of SSF scores, in both research and clinical practice.

Our first specific aim was to identify and suggest a strategy for handling items of the SSF that are only applicable to specific subsets of respondents. After careful review we chose to exclude items 23 and 33–38 from the SSF scoring and recommend that this strategy be employed for future studies using the SSF. How missing data is handled in research may have far‐reaching consequences, such as inflating the risk of bias in statistical analyses or misinterpreting variations in the data (Bennett, 2001). The decision to exclude items from the SSF was motivated both by the number of missing answers reported in previous studies and repeated to an even higher degree in our study, and by the nature of the items, all constituting real differences in experiences of parenthood, such as receiving a healthcare allowance, or caring for more than one child.

Our second specific aim was to improve the interpretability of SSF scores. This was achieved by using parents' self‐reports of recent help‐seeking behavior, experiences of mental ill‐health and experiences of parenthood as an anchor (Guyatt *et al*., 2002) from which to derive empirical cut‐offs that could be used to determine whether a respondent was currently more or less affected by parental stress. Using self‐reports as a criterion against which to validate scores on patient‐reported outcome measures (PROMs) may seem like a novel approach but has precedence in the literature (Button, Kounali, Thomas *et al*., 2015; Guyatt *et al*., 2002). Not only are self‐reports a practical way to “assign qualitative meaning” to, and hence improve the interpretability of scores (Mokkink *et al*., 2010, p. 743). Self‐reports may also be particularly useful when assessing internalized states (Craig *et al*., 2016), and may serve as a complement to standardized tests when the construct the instrument aims to measure is subjective, and hence may be interpreted differently by different individuals (Guyatt *et al*., 2002).

The credibility of the empirical cut‐offs was then tested by comparing score distribution around the cut‐offs for parents of children with and without developmental disabilities. In this study, a larger proportion of parents of children with developmental disabilities exceeded the cut‐offs for the SSF Stress, Strength and Risk scales while only a smaller proportion of parents of children without disabilities did so. This constituted our third specific aim, showing that the proposed cut‐offs were able to separate scores of known groups in a satisfactory way. However, we also found a considerable overlap between the SSF scores of parents of typically developing children and parents of children with developmental disabilities on each of the subscales. This aligns with previous research indicating that experiences of parental stress are subjective, and highly variable among parents (Fang *et al*., 2022), and that higher degrees of parental stress and lower degrees of strength are not universal experiences for parents of children with developmental disabilities (Hastings, 2016; Hoyle *et al*., 2021; Scherer *et al*., 2019).

Our final specific aim was to provide percentile values for SSF scores. This was achieved by using SSF scores from parents of children without developmental disabilities to construct a conversion chart, presented in Table S5. Percentiles are a common, and easily understandable, metric that can be used as a standardized basis when assessing test scores in both clinical practice, and in research (de Beurs, Boehnke & Fried, 2022). Access to a standardized way of interpreting scores on the SSF will enable healthcare professionals to conduct a more in‐depth evaluation of parental stress and strength in parents of children with developmental disabilities as compared to parents of children without disabilities. This may in turn facilitate discussions of parental support needs and parental engagement thus enabling healthcare professionals to adapt interventions to the needs of the child, the parent, and the family (Kokorelias *et al*., 2019; Popov *et al*., 2021). If the SSF is administered both before and after an intervention, the existence of percentiles, and empirical cut‐offs will enable a more reliable assessment of whether the intervention has benefited parental well‐being and led to a change in the parent's at‐risk status (Button *et al*., 2015; Guyatt *et al*., 2002).

## LIMITATIONS AND FUTURE STUDIES

The empirical cut‐offs are based on parent self‐reports of recent help‐seeking behavior, experiences of mental ill‐health and experiences of parenthood. These cut‐offs cannot, and should not, be used as indicative of a diagnosis but rather as a means to enhance the interpretability of the SSF (Guyatt *et al*., 2002). However, it is important to note that the empirical cut‐offs presented in this study are only relevant for the adapted SSF questionnaire, comprising 38 items instead of the original 45. Further, although careful steps were taken to prevent bias when categorizing parents' free text answers, the 20% of parent responses that were categorized after consensus discussion may be vulnerable to inter‐observer bias. It should also be acknowledged that relying on parents' self‐reports of their child's disability status entails a risk that parents are not fully informed of the disability status of their child (Carroll, 2013), which may lead to an under‐reporting of disability. This limitation primarily applies to reports of disability status in datasets A and B, and to a lesser degree to dataset C, consisting partly of parents of children in contact with habilitation services, that cater only to specific disabilities.

There were slight differences in reportable categories between datasets A, B, and C where the datasets A and B were highly similar, whereas dataset C featured older children and a different distribution of developmental disabilities (disabilities that qualify for reception of habilitation services). Together, however, the three datasets encompass parental experiences of impact of parenthood from early childhood to the end of middle school age, relating to the most common types of developmental disabilities. While this study represents experiences of parenthood related to a wide range of developmental disabilities, each group is too small to enable comparisons of parental experiences among different disabilities, making it difficult to discern whether impact on parents differs in degree, or across SSF‐domains, depending on the child's type of disability. There are indications that experiences of parental stress vary depending on the child's type of disability (Barroso *et al*., 2018; Craig *et al*., 2016). Hence, future studies should aim to investigate SSF score distribution of larger groups of parents of children with specific developmental disabilities.

The observation that SSF scores decrease slightly for parents of older typically developing children but remain relatively stable for parents of children with developmental disabilities may reflect an actual difference in experiences of parental stress between parents of school‐aged children with and without disabilities. However, it may also be explained by the different distribution of age and developmental disabilities between the datasets. Future studies investigating change in SSF scores over time and providing reference values for impact on parents of high school students with and without developmental disabilities may provide further insights into the relation between parental experiences of impact as measured by the SSF, and the child's age and type of disability.

## CONCLUSION

Parents' experiences of strength and stress are important to assess both when healthcare professionals collaborate with parents, and when evaluating the effectiveness of interventions aimed at either the child, the parents, or the family. In this study we suggest modifications to the SSF, to ensure that all items apply to all potential respondents. We provide empirical cut‐offs to each of the three subscales of the SSF to improve the interpretability of the questionnaire, and we present a chart for converting SSF scores into percentile values to facilitate the use of the SSF as an evaluation and outcome measure. We hope that these enhancements will help make sense of scores on the SSF, thus making the instrument more useful in clinical practice, and in research.

The authors wish to thank the parents who participated in this study. Author contributions are as follows: conceptualization: AS, SS, SB. Data curation: AS. Formal analysis: AS, SB. Methodology: AS, SS, SB. Project administration: AS, SB. Resources: SS, AS, SB. Supervision: AS, SS, CM, AEH, Validation: AS. Visualization: AS. Writing: SB, AS. Review, and editing: SB, AS, SS, CM, AEH. The R code utilized in this study can be provided by request to the corresponding author.

## Supporting information


**Figure S1.** ROC‐curves for the SSF stress, strength and risk‐scales.

**Figure S2.** Empirical cumulative distribution function for the SSF stress, strength and risk scales.

**Table S1.** Demographic composition of Dataset A (*n* = 129), with regards to children's age and condition.

**Table S2.** Demographic composition of Dataset B (*n* = 128), with regards to children's age and condition.

**Table S3.** Demographic composition of Dataset C (*n* = 319), with regards to children's age and condition.

**Table S4.** Categorization of the 129 respondents who could answer questions 1–3 listed in the main text.

**Table S5.** A conversion chart for a specific SSF scale value and the corresponding percentile among parents of children without any disability (*n* = 373). The corresponding cut‐offs from Table 3 are marked in bold.
