# Supplementary figures and images for: Interpretability and clinical utility of the strength and stressors in parenting questionnaire
Source: Scand J Psychol. 2024 Sep 16;66(1):141–9. doi: 10.1111/sjop.13073 (PMC11735247; doi:10.1111/sjop.13073)

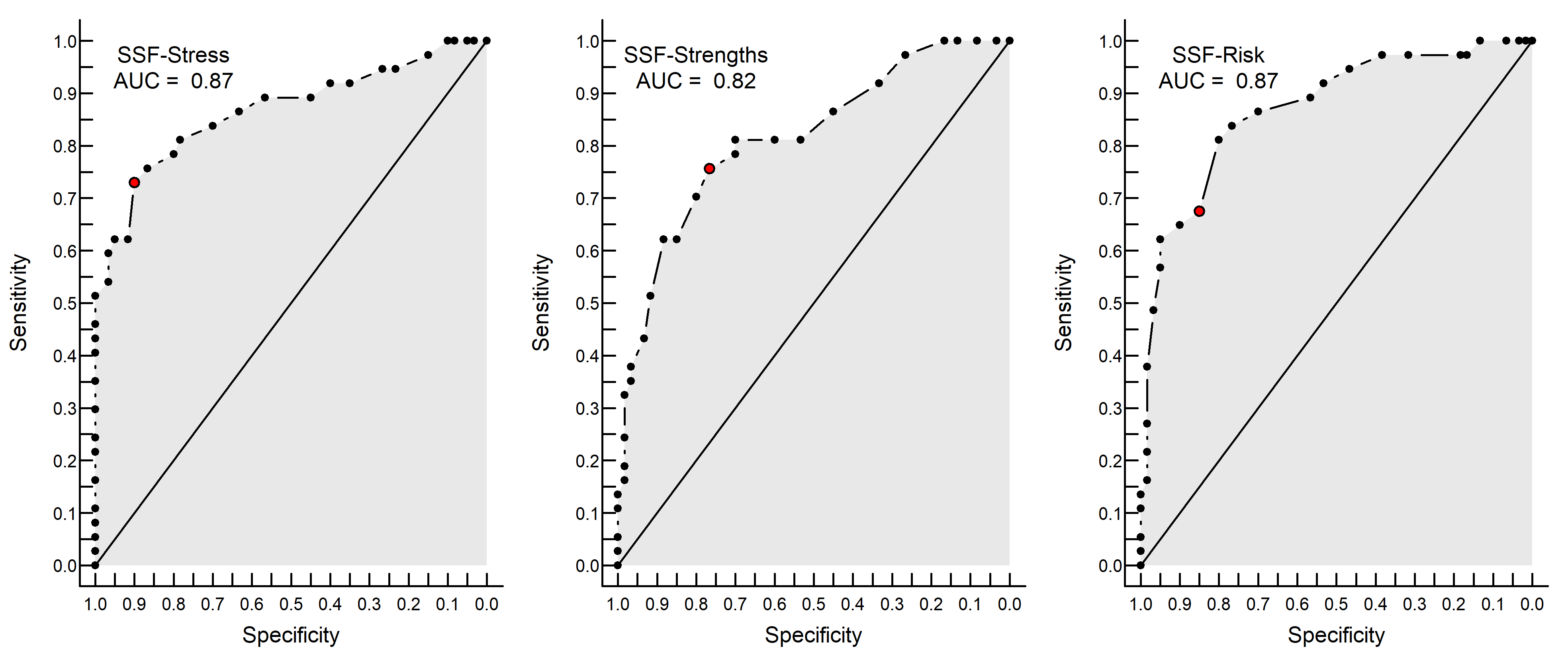

Supplement: Supplementary file 1 — Figure S1. ROC‐curves for the SSF stress, strength and risk‐scales. [file SJOP-66-141-s003.tiff]

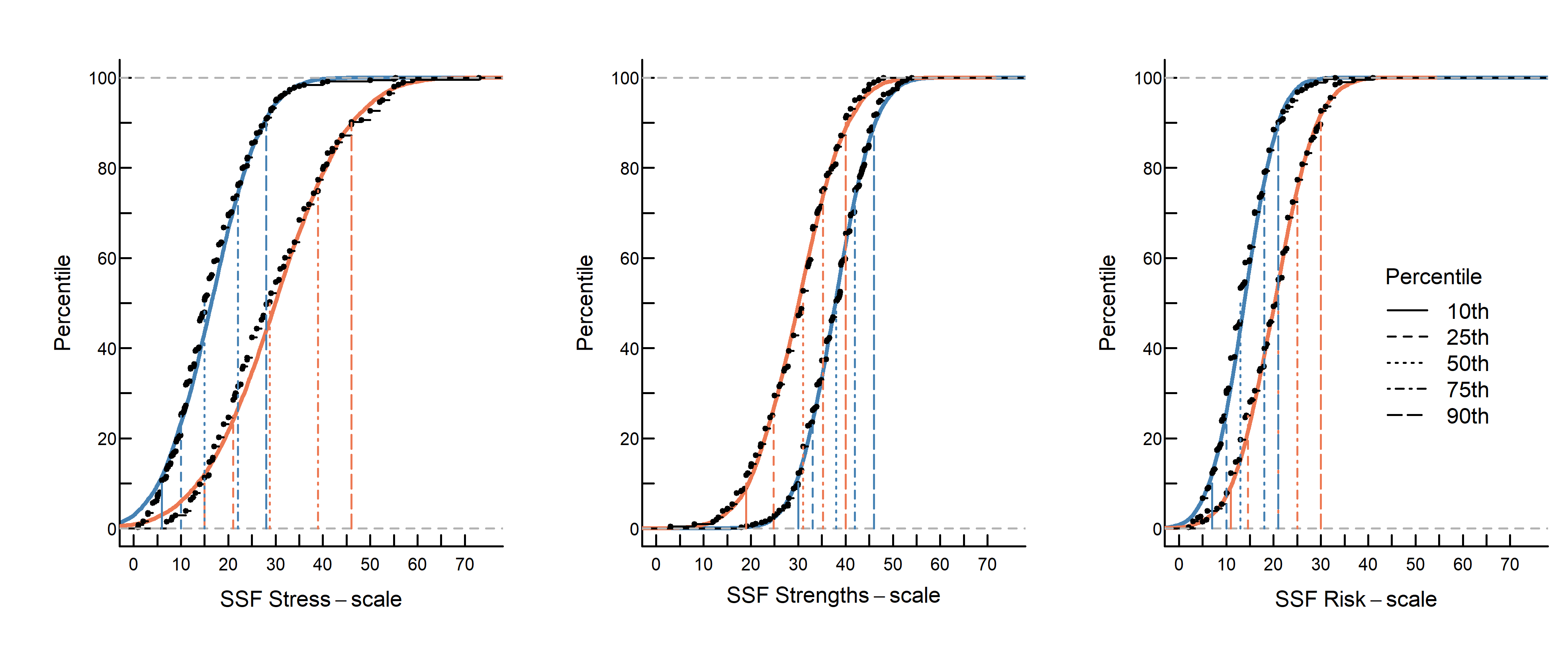

Supplement: Supplementary file 2 — Figure S2. Empirical cumulative distribution function for the SSF stress, strength and risk scales. [file SJOP-66-141-s004.tiff]
