# Supplementary material for: Interpretability and clinical utility of the strength and stressors in parenting questionnaire
Source: Scand J Psychol. 2024 Sep 16;66(1):141–9. doi: 10.1111/sjop.13073 (PMC11735247; doi:10.1111/sjop.13073)
Supplement: Supplementary file 4 — Table S2. Demographic composition of Dataset B (n = 128), with regards to children's age and condition. [file SJOP-66-141-s001.docx]

| **Table S2.** Demographic composition of Dataset B (*n* = 128), with regards to children’s age and condition. | | | | | | |
| --- | --- | --- | --- | --- | --- | --- |
| Child age (years) | No disability | Autism | ADHD | Intellectual disability | DLD | Motor disability |
| 2 | 8 |  |  |  |  | N/A |
| 3 | 23 | 1 | 1 |  | 2 | N/A |
| 4 | 16 | 2 | 1 |  | 14 | N/A |
| 5 | 22 | 2 |  |  | 6 | N/A |
| 6 | 10 | 2 | 4 | 1 | 15 | N/A |
| 7 |  | 1 | 1 |  | 3 | N/A |
| 8 |  |  |  |  | 1 | N/A |
| 9 |  |  |  |  | 2 | N/A |
| 10 |  |  |  |  | 1 | N/A |
| 11 |  |  | 1 |  | 1 | N/A |
| 12 |  |  |  |  |  | N/A |
| 13 |  |  |  |  |  | N/A |
| Total | 79 | 8 | 8 | 1 | 45 | N/A |
| *Note.* Conditions may co-occur in one individual child, which is why the sum in the total row adds up to more than the number of individuals. No information was available regarding motor disability. | | | | | | |
