# Supplementary material for: Interpretability and clinical utility of the strength and stressors in parenting questionnaire
Source: Scand J Psychol. 2024 Sep 16;66(1):141–9. doi: 10.1111/sjop.13073 (PMC11735247; doi:10.1111/sjop.13073)
Supplement: Supplementary file 5 — Table S3. Demographic composition of Dataset C (n = 319), with regards to children's age and condition. [file SJOP-66-141-s002.docx]

| **Table S3.** Demographic composition of Dataset C (*n* = 319), with regards to children’s age and condition. | | | | | | |
| --- | --- | --- | --- | --- | --- | --- |
| Child age (years) | No disability | Autism | ADHD | Intellectual disability | DLD | Motor disability |
| 2 | 0 |  | N/A |  | N/A |  |
| 3 | 0 |  | N/A |  | N/A |  |
| 4 | 0 |  | N/A |  | N/A |  |
| 5 | 0 |  | N/A |  | N/A |  |
| 6 | 1 |  | N/A |  | N/A |  |
| 7 | 38 | 16 | N/A | 3 | N/A | 4 |
| 8 | 45 | 8 | N/A | 6 | N/A | 7 |
| 9 | 36 | 8 | N/A | 3 | N/A | 8 |
| 10 | 33 | 11 | N/A | 1 | N/A | 7 |
| 11 | 33 | 18 | N/A | 7 | N/A | 4 |
| 12 | 19 | 2 | N/A | 1 | N/A |  |
| 13 | 0 |  | N/A |  | N/A |  |
| Total | 205 | 63 | N/A | 21 | N/A | 30 |
| *Note.* Conditions may co-occur in one individual child, which is why the sum in the total row adds up to more than the number of individuals. No information was available ADHD and/or DLD. | | | | | | |
