# Supplementary material for: Interpretability and clinical utility of the strength and stressors in parenting questionnaire
Source: Scand J Psychol. 2024 Sep 16;66(1):141–9. doi: 10.1111/sjop.13073 (PMC11735247; doi:10.1111/sjop.13073)
Supplement: Supplementary file 6 — Table S4. Categorization of the 129 respondents who could answer questions 1–3 listed in the main text. [file SJOP-66-141-s005.docx]

| **Table S4.** Categorization of the 129 respondents who could answer questions 1-3 listed in the main text. | | | | |
| --- | --- | --- | --- | --- |
| Sorting | Affected_freetext_ | Not Affected_freetext_ | Missing Q3 | Total |
| Yes-response to both Q1 and Q2 | 9 | 0 | 4 | 13 |
| Yes-response only to Q1 | 4 | 1 | 3 | 8 |
| Yes-response only to Q2 | 5 | 4 | 7 | 16 |
| No to Q1 and Q2 | 16 | 26 | 35 | 76 |
| Total | 34 | 31 | 49 | 113 |

*Note.* Sorting into Affected_freetext_ or not was based on the free-text answers to Q3. The 37 (13 + 8 + 16) respondents who answered Yes to Q1 or Q2 (or both) were sorted as “more affected” whereas the 15 who we labelled as Affected_freetext_ but did not reply yes to Q1 or Q2 were sorted as “less affected”. The remaining 61 (26 + 35) were sorted as “not affected”. Relevant cells are marked in grey.
