# Supplementary material for: Interpretability and clinical utility of the strength and stressors in parenting questionnaire
Source: Scand J Psychol. 2024 Sep 16;66(1):141–9. doi: 10.1111/sjop.13073 (PMC11735247; doi:10.1111/sjop.13073)
Supplement: Supplementary file 7 — Table S5. A conversion chart for a specific SSF scale value and the corresponding percentile among parents of children without any disability (n = 373). The corresponding cut‐offs from Table 3 are marked in bold. [file SJOP-66-141-s007.docx]

| **Table S5.** A conversion chart for a specific SSF scale value and the corresponding percentile among parents of children without any disability (n = 373). Marked in bold are the corresponding cut-offs from Table 3. | | | | | | | |
| --- | --- | --- | --- | --- | --- | --- | --- |
| SSF scale Value | Percentile on Stress | Percentile on Strengths | Percentile on Risk | SSF scale Value | Percentile on Stress | Percentile on Strengths | Percentile on Risk |
| 1 | 1 |  |  | 26 | 88 | 97 | 98 |
| 2 | 1 |  | 1 | 27 | 90 | 96 | 98 |
| 3 | 3 |  | 2 | 28 | 92 | 94 | 99 |
| 4 | 5 |  | 3 | 29 | 94 | 91 | 99 |
| 5 | 7 |  | 8 | 30 | 95 | 88 | 99 |
| 6 | 10 |  | 11 | 31 | 96 | 82 | 100 |
| 7 | 13 |  | 15 | 32 | 97 | 77 |  |
| 8 | 16 |  | 20 | 33 | 97 | 73 |  |
| 9 | 21 |  | 26 | 34 | 98 | 68 |  |
| 10 | 27 |  | 32 | **35** | 98 | **63** |  |
| 11 | 33 |  | 40 | 36 | 98 | 58 |  |
| 12 | 36 |  | 47 | 37 | 98 | 54 |  |
| 13 | 40 |  | 56 | 38 | 98 | 49 |  |
| 14 | 47 |  | 61 | 39 | 98 | 41 |  |
| 15 | 51 |  | 64 | 40 | 99 | 34 |  |
| 16 | 55 |  | 71 | 41 | 99 | 29 |  |
| 17 | 60 |  | 75 | 42 | 99 | 24 |  |
| 18 | 64 | 100 | 80 | 43 | 99 | 21 |  |
| 19 | 68 | 99 | 84 | 44 | 99 | 16 |  |
| **20** | 71 | 99 | **89** | 45 | 99 | 12 |  |
| 21 | 74 | 99 | 91 | 46 | 99 | 9 |  |
| 22 | 77 | 99 | 92 | 47 | 99 | 5 |  |
| 23 | 80 | 99 | 94 | 48 | 99 | 3 |  |
| 24 | 82 | 98 | 95 | 49 | 99 | 3 |  |
| **25** | **86** | 97 | 97 | 50 | 100 | 2 |  |
| *Note*. See below for example conversions.  A parent who reports a Stress score of 21 is similarly stressed as the 74^th^ percentile of stressed parents to children without disabilities. In other words, that parent is among the 26% *most stressed* parents.  A parent who reports a Strength score of 21 is similarly “strong” as the 99^th^ percentile of “weakest” parents to children without disabilities. In other words, that parent is among the 1% *least Strong* parents.  A parent who reports a Risk score of 18 is similarly at risk as the 80th percentile of at-risk parents to children without disabilities. In other words, that parent is among the 20% *most at-risk* parents. | | | | | | | |
